# European Network on Myalgic Encephalomyelitis/Chronic Fatigue Syndrome (EUROMENE): Expert Consensus on the Diagnosis, Service Provision, and Care of People with ME/CFS in Europe

**DOI:** 10.3390/medicina57050510

**Published:** 2021-05-19

**Authors:** Luis Nacul, François Jérôme Authier, Carmen Scheibenbogen, Lorenzo Lorusso, Ingrid Bergliot Helland, Jose Alegre Martin, Carmen Adella Sirbu, Anne Marit Mengshoel, Olli Polo, Uta Behrends, Henrik Nielsen, Patricia Grabowski, Slobodan Sekulic, Nuno Sepulveda, Fernando Estévez-López, Pawel Zalewski, Derek F. H. Pheby, Jesus Castro-Marrero, Giorgos K. Sakkas, Enrica Capelli, Ivan Brundsdlund, John Cullinan, Angelika Krumina, Jonas Bergquist, Modra Murovska, Ruud C. W. Vermuelen, Eliana M. Lacerda

**Affiliations:** 1 London School of Hygiene and Tropical Medicine, Faculty of Infectious and Tropical Diseases, London WC1E 7HT, UK; 2 BC Women’s Hospital, Vancouver, BC V6H 3N1, Canada; 3 Faculty of Medicine Créteil—Paris Est, 94010 Creteil, France; fj.authier@gmail.com; 4 Institute of Medical Immunology, Charité-Universitätsmedizin Berlin, 10117 Berlin, Germany; Carmen.Scheibenbogen@charite.de; 5 Neurology and Stroke Unit—Neuroscience Department—A.S.S.T.—Lecco, 23900 Merate, Italy; lorusso.lorenzo@gmail.com; 6 National Advisory Unit on CFS/ME, Oslo University Hospital, Rikshospitalet OUS, 0372 Oslo, Norway; ihelland@ous-hf.no; 7 Chronic Fatigue Unit, Hospital Universitari Vall d’Hebron University Hospital (VHIR), Universitat Autònoma de Barcelona, E-08035 Barcelona, Spain; jalegre@vhebron.net; 8 Central Military Emergency University Hospital, Titu Maiorescu University, 040441 Bucharest, Romania; sircar13@yahoo.com; 9 Institute of Health and Society, Medical Faculty, University of Oslo, Box 1089 Blindern, 0317 Oslo, Norway; a.m.mengshoel@medisin.uio.no; 10 Bragée ME/CFS Center, 115 26 Stockholm, Sweden; olli.polo@unesta.fi; 11 Department of Pediatrics, School of Medicine, Technical University of Munich, 80333 Munich, Germany; uta.behrends@mri.tum.de; 12 Privat Hospitalet Danmark, 2920 Charlottenlund, Denmark; hnreum@dadlnet.dk; 13 Department of Hematology, Oncology and Tumor Immunology, Institute for Medical Immunology, Charite Medical School, 10117 Berlin, Germany; patricia.grabowski@charite.de; 14 Medical Faculty Novi Sad, University of Novi Sad, 21000 Novi Sad, Serbia; slobodan.sekulic@mf.uns.ac.rs; 15 Centre of Statistics and Its Applications, University of Lisbon, 1749-016 Lisbon, Portugal; Nuno.Sepulveda@lshtm.ac.uk; 16 Erasmus MC University Medical Center, 3015 Rotterdam, The Netherlands; fer@estevez-lopez.com; 17 Department of Hygiene, Epidemiology, Ergonomics and Postgraduate Education, Nicolaus Copernicus University in Torun, Collegium Medicum, 85-067 Bydgoszcz, Poland; p.zalewski@cm.umk.pl; 18 Society and Health, Buckinghamshire New University (retired), High Wycombe HP11 2JZ, UK; derekpheby@btinternet.com; 19 Division of Rheumatology, ME/CFS Research Unit (Lab 009–Box 02), Vall d’Hebron Hospital Research Institute (VHIR), Val d’Hebron Hospital Research Unit (VIHR), Passeig de la Val d’Hebron 119-129, E-08035 Barcelona, Spain; jesus.castro@vhir.org; 20 Department of PE and Sports Science, University of Thessaly, 421 00 Trikala, Greece; gsakkas@med.uth.gr; 21 Department of Earth and Environmental Sciences, University of Pavia, 27100 Pavia, Italy; enrica.capelli@unipv.it; 22 Department of Regional Health Research, University Hospital of Southern Denmark, 5000 Odense, Denmark; Ivan.Brandslund@rsyd.dk; 23 School of Business & Economics, National University of Ireland Galway, University Road, H91 TK33 Galway, Ireland; john.cullinan@nuigalway.ie; 24 Department of Infectiology and Dermatology, Riga Stradins University, LV-1067 Riga, Latvia; angelika.krumina@rsu.lv; 25 Department of Chemistry—Biomedical Center, Analytical Chemistry and Neuro Chemistry, Uppsala University, 751 23 Uppsala, Sweden; jonas.bergquist@kemi.uu.se; 26 The Myalgic Encephalomyelitis/Chronic Fatigue Syndrome (ME/CFS) Collaborative Research Centre, Uppsala University, 751 23 Uppsala, Sweden; 27 Institute of Microbiology and Virology, Riga Stradins University, LV-1067 Riga, Latvia; Modra.Murovska@rsu.lv; 28 CFS/ME Medical Centre, 1078 Amsterdam, The Netherlands; rv@cvsmemc.nl; 29 Department of Clinical Research, Faculty of Infectious and Tropical Diseases, London School of Hygiene and Tropical Medicine, Keppel Street, London WC1E 7HT, UK; Eliana.Lacerda@lshtm.ac.uk

**Keywords:** Myalgic Encephalomyelitis/Chronic Fatigue Syndrome, diagnosis, health services, care

## Abstract

Designed by a group of ME/CFS researchers and health professionals, the European Network on Myalgic Encephalomyelitis/Chronic Fatigue Syndrome (EUROMENE) has received funding from the European Cooperation in Science and Technology (COST)—COST action 15111—from 2016 to 2020. The main goal of the Cost Action was to assess the existing knowledge and experience on health care delivery for people with Myalgic Encephalomyelitis/Chronic Fatigue Syndrome (ME/CFS) in European countries, and to enhance coordinated research and health care provision in this field. We report our findings and make recommendations for clinical diagnosis, health services and care for people with ME/CFS in Europe, as prepared by the group of clinicians and researchers from 22 countries and 55 European health professionals and researchers, who have been informed by people with ME/CFS.

## 1. Introduction

### 1.1. Standardization of Clinical Procedures and Services for Myalgic Encephalomyelitis/Chronic Fatigue Syndrome (ME/CFS) in Europe: The Origins

Initially designed by a group of ME/CFS researchers and health professionals, the European Network on Myalgic Encephalomyelitis/Chronic Fatigue Syndrome (EUROMENE) has received funding from the European Cooperation in Science and Technology (COST)—COST action 15111 [1] —from 2016 to 2020. The main goal of the Cost Action was to assess the existing knowledge and experience on health care delivery for people with Myalgic Encephalomyelitis/Chronic Fatigue Syndrome (ME/CFS) in European countries and to enhance coordinated research and health care provision in this field.

One of the aims of the network was to *define a standardised clinical diagnosis for ME/CFS for clinical and research use.* With the paucity and lack of integration of clinical guidelines in European countries [1], a high need has been identified for addressing the uncertainties around diagnosis and treatment, and to support the development of health services and standard clinical practices for people with ME/CFS across the continent. We report here on the recommendations for clinical diagnosis and management of ME/CFS in Europe, as prepared by the group of clinicians and researchers from 22 countries participating in the network activities (including on Near Neighbour Country–Belarus), and 55 European researchers and health professionals, who have been informed by people with ME/CFS. The participating countries are Austria, Belarus, Belgium, Bulgaria, Denmark, Finland, France, Germany, Greece, Ireland, Italy, Latvia, Netherlands, Norway, Poland, Portugal, Romania, Serbia, Slovenia, Spain, Sweden, United Kingdom. The researchers’ names and affiliations are listed in the COST Action website [1]. 

### 1.2. The Population Burden of the Disease and the Need for Better Recognition

ME/CF is characterised by intolerance to efforts expressed by profound or pathological fatigue, malaise, and other symptoms aggravated by physical or cognitive efforts at intensities previously well tolerated by the individual. Intolerance to efforts may be experienced immediately or typically be delayed for hours or a day or more after exertion and is associated with slow recovery. This marked and prolonged exacerbation of symptoms of ME/CFS, which follows physical activity and, in some cases, cognitive activity, is termed post-exertional malaise (PEM) and may last several days.

Other key symptoms include unrefreshing sleep, cognitive impairment, orthostatic intolerance, and pain, including muscle and joint pain and headaches. The symptoms are persistent or recurrent over long periods of time and lead to a significant reduction in previous levels of functioning. Diagnosis is clinical, owing to the absence of biomarkers, and based on detailed clinical history and physical examination by a competent clinician [2,3,4,5]. There is no causal treatment for the disease. With symptom-oriented support, there can be improvement with time, or patients may learn to manage their illness. There is little evidence on long term prognosis. However, full recovery is not the norm, particularly in adults [2,3,4,5,6,7], and in addition, there has been a small number of studies reviewing mortality among people with ME/CFS. These are consistent in demonstrating increases in mortality from suicide, in the UK [8] and in the US [9,10]. One American study demonstrated increased cardiovascular and cancer mortality [11], but this has not been confirmed by other studies.

Prevalence rates have been estimated as between 0.1 and 0.7%, and the incidence rate as 0.015 new cases/1000-year [12]. This could represent between 1 million and over 5 million people, probably around 3 million, in the European continent living with ME/CFS. However, there are no European-wide estimates of disease burden [13]. A much larger number of people will have chronic fatigue for other reasons, and many of them will also be significantly incapacitated. At least 2/3 of the cases are in women [12,13], with young people in their most productive phases of life being preferentially affected. However, ME/CFS has been reported in all age groups [14,15]. Quality of life of those with ME/CFS is on average lower than with other chronic or disabling diseases, such as multiple sclerosis [16], cancer, rheumatoid arthritis, depression [17], diabetes, epilepsy, or cystic fibrosis [18]. Economic costs are considerable [19,20,21,22,23] with repercussions for the individuals affected, their families and society, as well as to educational and occupational services. 

Many will be unable to work or do so only on a part-time basis; with some in the milder spectrum of the disease able to work full time, however, often at the cost of enduring significant symptoms and sacrificing their social life and other interests due to the need to rest when not working [24,25]. In the absence of economic analysis on the costs of the disease in Europe, we estimate, based on data from the UK, ME/CFS may cost some 40 billion Euros per year to health services and society [22]. There is, however, a large degree of imprecision in these estimates, due to variation in coverage and costs of health services provision and living costs across the Continent, as well as in many cases failure to recognize or diagnose the disease, which not only contributes to this imprecision but also may result in patients being treated inappropriately through their being misdiagnosed as having a psychiatric illness.

Despite the substantial disease burden, the health needs of people with ME/CFS remain largely unmet in Europe, as in many other parts of the world. Clinical services for people with the disease are in small numbers and sparse. A large proportion of the population with the disease has very limited access to health services, including in the public, mixed, and private sectors, because of either geographical inaccessibility, disability, or unsympathetic responses from healthcare professionals. The still limited knowledge of health professionals about the disease, including those in primary care, who are often the first port of call for those with ME/CFS, means diagnosis is often missed or delayed, and not infrequently patients remain undiagnosed and do not receive appropriate care for long periods of time. While waiting for diagnosis, patients often encounter difficulties in getting help from the health and other services, and their suffering and needs are not fully recognised, not only by health professionals but also by employers and educators. On the other hand, on some occasions, patients are over-investigated, with inherent risks and unnecessary costs to individuals and society. People with ME/CFS may easily get trapped into a situation where, while unable to carry on or start meaningful work- or school-related activities, they receive very little guidance from the health sector or support from social services—where they feel disbelieved and neglected and are often failed by the welfare system [24]. Their disability contributes to social isolation, which adds to their burden, and limits their chances of recovery or re-integration in society, by restricting access to healthcare and social support.

## 2. Methods

### 2.1. Development of Recommendations

The EUROMENE network activities were organised in Working Groups (WG), including the Clinical Group, tasked to explore existing methods used for the diagnosis of cases in Europe and to develop recommendations for the diagnosis and treatment of people with ME/CFS in the continent. The recommendations for standardising the diagnostic criteria for ME/CFS to be used by European researchers are covered in a related EUROMENE document [26], which will allow comparability and better estimates.

We have not systematically reviewed the evidence in relation to diagnostic criteria and interventions, as this has been done by others. Thus, the following recommendations are pragmatic and were based on the working group member’s collective and consensual assessment of key documents on clinical definitions of ME/CFS [2,4,5,6,27,28] and existing studies and guidelines for clinical assessments and care used in Europe and internationally [1]. The WG members met on various occasions (WG meetings) to agree on key documents and to consider them, based on the members’ experiences and expertise and relevance for clinical practice in Europe. We recognise that there is still limited evidence-based research on ME/CFS; as we witness progresses in this field, we recognise the need for frequent reviews of these recommendations, in line with emerging evidence.

### 2.2. Considerations on ME/CFS Diagnosis for Clinical Purposes

Many diagnostic criteria have been proposed for use in clinical practice, of which those by the Institute of Medicine (currently, National Academy of Medicine), known as the IOM criteria, have received international recognition. Their relative simplicity makes them ideal for use in primary care. An editorial in the Lancet in 2015 [29] suggested that the adoption of the new name Systemic Exertion Intolerance Disease (SEID) could lead to changed attitudes and greater acceptance of the condition. However, despite the fact that the term ME/CFS implies a particular underlying pathology which has yet to be demonstrated, this remains the most widely used diagnostic term, and it is arguable also that SEID understates the severity of the condition, since exertion intolerance is by no means its only clinical feature. The term ME, an abbreviation for myalgic encephalomyelitis, in particular, is unsatisfactory as it suggests that the pathological process underlying the disease is an inflammatory process affecting the brain. There is a lack of convincing evidence for this, and the truth is undoubtedly both more arcane and more complex. The term CFS, short for chronic fatigue syndrome, is equally unsatisfactory, as it implies that fatigue is the main symptom of the illness, whereas in fact its clinical features are far more wide-ranging. The composite term ME/CFS thus carries the disadvantages and shortcomings of both contributary terms. We use it and recommend its use, on purely pragmatic grounds despite these problems, since most clinicians and researchers working in the field use the term and understand its shortcomings. It is part of the lingua franca and enables those working in the field to communicate effectively with each other and, it is to be hoped, to make progress which will enable us ultimately to establish the underlying pathology with greater precision, leading in turn to the development of more appropriate and accurate terminology.

A case of ME/CFS in an adult patient requires the presence of symptoms for at least 6 months and are typically present for at least half of the time (Box 1).

Box 1. **Institute of Medicine (IOM) criteria for the diagnosis of ME/CFS.**

Required symptomsSubstantial reduction or impairment in the ability to engage in pre-illness levels of activity (occupational, educational, social, or personal life) with profound fatigue of new onset, which is present for at least 6 months, is not explained by ongoing or unusual excessive exertion and is not substantially relieved by restPost-exertional malaise (PEM)Unrefreshing sleepAt least one of the following: Cognitive impairmentOrthostatic intolerance

For full details, see Institute of Medicine (IOM), 2015 [6].

The Canadian Consensus Criteria (CCC) are particularly suitable for diagnosis confirmation and case sub-grouping in secondary care, as well as in research (Box 2). The CDC-1994/Fukuda et al. criteria [27] may also be used as a screening tool for diagnosis in clinical practice, but we recommend that only cases with post-exertional malaise (PEM) (which is optional in that definition), are included for diagnosis (Box 3). Note that although the CDC-1994 criteria have been developed for research purposes, they have often been used for diagnosis purposes in clinical practice and are still a preferred case definition by some in Europe.

For children, the IOM [6] and Rowe et al., 2017, criteria [4] (Box 4) may be used. The latter is based on 6 cardinal paediatric symptoms and a disease duration of 6 months; a diagnosis of “postinfectious fatigue syndrome” (PFS) is made when the symptoms are present for 3 months following an acute infection. The Canadian Consensus criteria [2] may also be used in children, as proposed by Jason et al. [5,30]. However, using 3 months of symptoms is sufficient for diagnosis in children and adolescents.

Diagnosis in both adults and children can be suspected earlier, and the primary care physician should be proactive in starting diagnostic investigations. Initial management and referral may be considered when diagnosis is suspected or with 3 months of symptoms, as appropriate.

Box 2. **Canadian Consensus Criteria for the diagnosis of ME/CFS.**

The required symptoms, listed below, must be persistently or recurrently present for at least 6 months in adults (3 months in children and adolescents). If other conditions have the same symptoms, those conditions must be assessed and treated optimally first before a diagnosis of ME/CFS can be made. Exclusionary conditions should be ruled out by a combination of clinical history, physical examination, and complementary tests.Pathological fatiguePost-exertional malaise and worsening of symptomsSleep dysfunctionPainCognitive symptoms (at least two symptoms from a list provided)In addition, at least one symptom from two from the following categories of symptoms are required:AutonomicNeuroendocrineImmune

For full details, see Carruthers et al., 2003 [2]. The structure of the CCC definition in adults and some aspects of the CDC-1994 [26] criteria were used to create a paediatric cases definition of ME/CFS [5,30].

Box 3. Modified* CDC-1994 Criteria for the diagnosis of ME/CFS.

Primary symptoms Clinically evaluated, unexplained, persistent, or relapsing chronic fatigue that is:Of new or definite onset (has not been lifelong);Is not the result of ongoing exertion;Is not substantially alleviated by rest;Results in substantial reduction in previous levels of occupational, educational, social, or personal activities;Is associated with post-exertional malaise (PEM)*.
Additional symptoms The concurrent occurrence of three or more of the following symptoms:Substantial impairment in short-term memory or concentration;Sore throat;Tender lymph nodes;Muscle pain;Multi-joint pain without swelling or redness;Headaches of a new type, pattern, or severity;Unrefreshing sleep.
These symptoms must have persisted or reoccurred during 6 or more consecutive months of illness and must not have started before the fatigue. * Modified for use in clinical diagnosis of ME/CFS, to include PEM as compulsory symptom (EUROMENE recommendation). Source: Fukuda et al. 1994 [27].

Box 4. **Paediatric diagnosis of ME/CFS.**

A diagnosis is based on persistent symptoms as below: Compulsory symptoms: Impaired functionPost-exertional symptomsFatigue
In addition, 2 of 3 groups of symptoms are required:Sleep problemsCognitive problemsPain
A diagnosis is made if all the criteria below apply:
Symptoms are persistent for 6 months (or for 3 months if post-infection) and at least some occur daily and are at least of moderate severityOther diagnoses are excluded by history, physical examination, and medical testing, including learning disabilitiesSeverity of symptoms over a pre-determined cut-off score

For full details, see Rowe et al., 2017 [4]. For research we recommend using the DePaul Symptom Questionnaire Pediatric (DSQ-Ped) [5].

## 3. Approach to the Diagnosis and Characterisation of Patients

### 3.1. Steps to Recognising ME/CFS Cases in Clinical Practice

#### Clinical History

History reveals the main symptoms, including extreme fatigue, fatigability, and cognitive difficulties that are worsened by physical or mental effort. Physical fatigue is often expressed as “lack of energy or stamina”, profound tiredness, or general weakness (Box 5).

Mental fatigue is expressed as cognitive problems, such as slowness of response, attention, and concentration problems; they are often referred by patients as “brain-fog” and result in reduced ability to perform “mental tasks”.

There is significant intolerance to efforts, both physical and mental, with post-exertional aggravation of symptoms or PEM. PEM typically has delayed onset, often noticed hours later or the following day, and lasts for variable and often extended periods of time—e.g., from a day in milder cases to many days or weeks in moderately and severely affected individuals.

Sleep is characteristically “non-restorative” or “unrefreshing”, and difficulty in initiating or maintaining sleep is common.

Orthostatic intolerance may be manifested with light-headedness and worsening of symptoms (such as fatigue, malaise, dizziness, nausea, palpitations) when assuming or persisting in the upright position for some time, usually a few minutes, but it may happen very soon after raising from the recumbent position or within up to 10 min or more, depending on severity of the dysautonomia. The most severely affected may be unable to stand for more than a few seconds.

Pain can be generalised and referred to joints, muscles, and adjacent soft tissues, with frequent headaches commonly reported. Pain may be migratory and variable in nature and is not associated with signs of inflammatory arthritis or myositis, with typical absence of joint swelling or redness.

There is considerable symptom overlap between ME/CFS and fibromyalgia [30], and a concomitant diagnosis of fibromyalgia [31,32] is often made. The latter requires pain to be generalised (present in at least 4 of 5 body regions) and is widespread and accompanied by other symptoms, such as fatigue, poor sleep, and cognitive difficulties [33].

Importantly, the symptoms of ME/CFS lead to substantial reductions in previous levels of activity and function. Some individuals will still manage full-time work or education, at least for some time. However, very often patients are unable to take up or continue full-time work or education, or any at all, with a significant minority (often quoted as corresponding to 25% of all patients) virtually home- or bedbound. Educational, social, and economic consequences take their toll, with a resulting compromise in emotional wellbeing.

Box 5. **Symptoms and complaints to consider when taking a clinical history.**

*Key symptoms*Persistent, debilitating symptoms that include extreme fatigue or lack of energy, assessed by the impairment in the ability to work, study, or undertake domestic tasks, leisure activities, and social interactions.Persistent exhaustion or unusually high levels of fatigue, aggravated by low levels of exertion, still, upright position, and stress (physical or emotional, such as infections or raised anxiety levels).Post-exertional malaise, or post-exertional exacerbation of symptoms: any or all symptoms can get worse following physical or mental efforts and stress—this can happen immediately or more typically delayed after a period following the exertion, e.g., which may be longer than 24 h; recovery to previous levels of functioning and symptom severity may last long (typically from a day to weeks).Sleep dysfunction with unrefreshing sleep, i.e., waking up not feeling rested as one would expect following a good night’s sleep.Complaints of cognitive impairment, such as poor memory, attention, and concentration, slow thinking, reasoning difficulties, sense of disorientation, or “brain fog”.Pain: muscle and joint pains, which may affect multiple sites and be migratory, but without local signs of inflammation; headaches (tension or migraine type); existing musculoskeletal symptoms may worsen.*Additional symptoms*Orthostatic intolerance, defined by symptoms occurring only or worsened in the upright position (particularly when not associated with movement—i.e., in the still position), and improved by lying down, e.g., palpitations, tremors, light-headedness, dizziness, weakness, nausea.Over-sensitivity to stresses and sensory stimuli such as light, noise, temperature changes, or touch.Intolerance to dietary and environmental factors, such as to alcohol, selected or multiple food intolerances and medications, new allergies.Infection-like immune symptoms, e.g., frequent and prolonged symptoms of upper respiratory tract infections, such as flu-like symptoms, tender cervical lymph nodes, sore throat, congested nose, shortness of breath.Symptoms of irritable bowel syndrome.Weight loss or gain.Sicca-symptoms (dry eyes, mouth, or the opposite: hypersalivation).Emotional instability, anxiety, and depression.*Symptoms’ characteristics* • Symptoms may start following infectious or other insults or insidiously. These are persistent, but they may fluctuate from day to day or during the day. Some people experience temporary partial remission of symptoms, which is followed by recurrence and may occur after physical or mental exertion beyond their tolerance level. Although *specific symptoms* vary in presentation and severity, the symptoms tend to follow a typical pattern of inter-relatedness. This means that patients may have difficulties in distinguishing whether their symptoms arise from lack of energy, pain, or sleep deprivation, for example. Fatigue and intolerance to efforts are key symptoms which are not always easy to interpret *Fatigue* is a main symptom, but its description and interpretation are variable. It usually represents a feeling of intense lack of physical energy or stamina and mental tiredness (reduced mental clarity with slowness in thinking and difficulty in understanding and processing information, focusing attention and forgetfulness), which restricts the ability to undertake physical and mental activities.*Intolerance to efforts is a key symptom, which relates to disease severity and previous levels of functioning.* The most severely affected may be limited in simple movements in bed, speaking or engaging in conversation, eating, and activities of daily living such as going to bathroom, bathing, showering, or dressing), milder cases who were previously very active (e.g., athletes) may remain active, though much less than previously.

### 3.2. Clinical Examination

- General physical examination may be entirely normal. However, some patients present with general aspect of tiredness or of being unwell. Nutritional status is usually satisfactory, though overweight or obesity may result from long-term inactivity or as a neuro-endocrine manifestation of the disease. On the other hand, signs of weight loss or low body mass index (BMI) may be present, more commonly in severely affected patients, although they may also raise suspicion of other severe morbidity; signs of neglect or poor care with basic needs, if noticed, should raise concerns about the wellbeing of the patient. Paleness and cold extremities may be noted.
- Orientation and cognition; patients are oriented, but they may show signs of slow thinking, poor attention and short memory and be lost for words; long consultations may elicit increasing cognitive and physical difficulties as the patient tires; on the other hand, some patients may show signs of anxiety and “wired-tiredness”, where they are restless in spite of being very tired physically and mentally. Emotional responses may be triggered as patients go through their histories and common difficulties experienced with their symptoms and lack of validation of their diagnosis and degree of disability, which are often not obvious to the untrained observer. In general, patients are highly motivated and willing to do whatever may be needed to improve their symptoms. However, secondary anxiety and depressed mood may be observed, and lack of motivation or despondency should raise the possibility of associated low mood.
- Skin: Paleness and cold extremities may be noted, often aggravated by upright position, which may be associated with low peripheral perfusion or autonomic dysfunction. Redness of lower extremities when sitting or standing may also be noted as a consequence of venous congestion.
- Head and neck: Enlarged lymph nodes may be noted especially on the neck and might be tender; non-exudative pharyngitis might be observed, and crimson crescents in the oral pharyngeal region have often been described [34].
- Chest and cardio-vascular: Examination of the lungs and heart is usually unremarkable, except for possible changes in heart rate and blood pressure. Mild regular tachycardia may be present at rest. Postural tachycardia (standing heart rate of >30/min above normal in patients older than 20 years and > 40/min above normal in younger patients, compared to lying down or >120 standing heart rate at any age) may happen immediately or within 10 min or more after standing up from the recumbent or sitting position; it may result from dysautonomia or relative hypovolemia and result in the diagnosis of postural tachycardia syndrome (POTS). Some patients develop hypotension upon standing, sometimes after a brief period of raised blood pressure. These signs are more common in the young and in some over-medicated patients and may be associated with postural hyperaemia or cold extremities.
- Abdomen: General standard examination is conducted to rule out other explaining diseases; mild diffuse abdominal tenderness is not uncommon.
- Musculoskeletal: Joints appearance is usually normal (no oedema or redness); tenderness of joints and soft tissues may also be present. Some patients have hypermobile joints or fulfil the clinical criteria of hypermobile Ehlers–Danlos syndrome (hEDS) [35,36], which should be recognized as a comorbidity.

Brief neurological examination: This is usually normal, muscle fatiguability is shown by lower handgrip strength compared to healthy individuals or by a rapid fall in grip strength measures during repetitive muscle contractions, particularly in severely affected cases [37]. Sensory examination may be normal, though hyperalgesia or allodynia may be present. Cognitive difficulties and the occasional fasciculation may be noticeable [38]. Brisk symmetrical reflexes in arms and legs may be observed. Cranial nerve examination is usually normal; however, pupil reaction might be slow. Subtle gait abnormalities may be associated with a feeling of instability, although a full-blown Romberg sign at examination is atypical [39]. A brief psychiatric assessment may show signs of associated anxiety or mood disorders or the presence of an alternative diagnosis. Signs suggestive of specific neurological or psychiatric abnormalities should be investigated further.

- In the more severely affected, signs of frailty may be evident; patients may be virtually bed-bound, sit in a wheelchair, have a pale and puffy face, have cold extremities, and not be able to remain or feel very uncomfortable in the upright position for longer than a few seconds or minutes. There is a general sense of weakness and lack of stamina, and short periods of break during clinical assessment may be required as the patient becomes visibly tired and shows signs of increasing cognitive difficulty. Symmetrical reduction in limb muscle strength may be observed on formal neurological examination, and the hand grip manometer will usually show reduced power, with decreasing values on repeated measurements.

### 3.3. Differential Diagnosis

Since fatigue is a common complaint in daily life and in association with a range of medical problems, it is important to note that most people with ongoing fatigue do not have ME/CFS but rather have symptoms that are caused by other conditions, emotional well-being, or life-style-factors. The presence of PEM, however, raises the level of suspicion, as this is quite typical, though not specific of ME/CFS.

The list of co-morbid conditions and differential diagnoses is not exhaustive. Examples are listed in Box 6 and Box 7. Some conditions are often present concomitantly to ME/CFS (co-morbidities). Other conditions may potentially exclude a diagnosis if they fully or mainly explain the symptoms. However, such conditions may also be co-morbid when their presence does not explain most of the symptoms and signs observed. In general, when one of these conditions is present and is not well-controlled, the patient should be offered optimum treatment and stabilization, before a diagnosis of ME/CFS is considered. Severe conditions should be explored early and excluded or treated promptly. Action is prompted by clinical suspicion and red flags, such as unintentional weight loss, prolonged fever ≥ 38 °C, persistently elevated inflammatory markers, significant abnormalities in physical examination, or suicidal ideation. Box 8 includes suggested diagnostic sub-categories, which may change as the clinical picture and further clinical and related information arise. It should be noted also that some comorbid conditions occur largely in the presence of ME/CFS and so their presence or otherwise should be noted when considering whether the possible comorbid condition fully explains the patient’s symptoms.

Box 6. **Co-morbid conditions which do not exclude ME/CFS diagnosis.**

FibromyalgiaRestless legs syndrome, periodic limb disorderPostural orthostatic tachycardia syndrome (POTS)Neuro-mediated hypotensionIrritable bowel syndromeFood intolerances and atopic conditionsMild anxietyMild depression

Hypermobility Ehlers–Danlos syndromeMyofascial pain syndromeSmall fibre neuropathySicca symptomsChronic pelvic pain, endometriosisInterstitial cystitisHashimoto thyroiditis; hypothyroidism controlled clinically)MigraineMast cell activation disorder, eosinophilic esophagitis

Box 7. **List of diseases where fatigue may be a prominent feature, which may preclude a diagnosis of ME/CFS if the disease largely explains the symptoms. They may, however, be co-morbidities with ME/CFS if they do not fully explain symptoms characteristic of ME/CFS (including fatigue, cognitive complains, sleep dysfunction, PEM).**

HypothyroidismHyperthyroidismMalignancyRheumatoid arthritis, systemic lupus erythematosus, polymyositis, Sjogren syndrome, psoriasis arthritisCrohn’s disease, ulcerative colitis, coeliac diseasePost-concussion syndrome, post-ICU syndrome, post-traumatic stress disorderHeart disease, such as heart failureSevere chronic obstructive pulmonary disease, other severe respiratory diseasesSevere anaemia, vitamin B12 deficiency, haemochromatosisRenal failureDiabetes mellitusAddison’s or Cushing’s disease, hyperparathyroidism, and other endocrine disordersBipolar disorder, schizophrenia, major depression, anorexia, bulimia, autismMultiple sclerosis, myasthenia gravis, other neuroimmunological diseases, paraneoplastic syndromesParkinson’s disease, Alzheimer’s disease, stroke, other serious neurodegenerative diseasesSleep apnoeaNarcolepsyHepatitis, tuberculosis, HIV/AIDS, neuroborreliosis, other chronic infectionsExcessive consumption/abuse of alcohol or other substances

### 3.4. Detailed Clinical Characterization, Laboratory, and Other Tests

Further patient characterization may involve the use of standard questionnaires—which may be self-completed or applied by an interviewer, and physical measures, which are used to assess function and disease severity. They are useful for patient’s baseline evaluation, and, when repeated subsequently, they provide indicators of disease course and evaluation of response to treatment. Core assessments shown in Box 8 include examples of tests that may be used routinely for that aim. When research studies are linked to clinical practice, these and other questionnaires and instruments may also be used [26]. Further laboratory tests and imaging studies may be needed to identify potential co-morbidities, and/or to exclude other diagnoses. These should be guided by clinical assessment and the need to exclude conditions that may explain the symptoms.

Examples of useful screening tests for initial investigations in primary care include full blood count, ferritin, liver enzymes, renal function, thyroid function, high-sensitivity C reactive protein (CRP) or erythrocyte sedimentation rate, electrolytes including sodium, potassium, calcium, inorganic phosphate, creatine phosphokinase (CK), and fasting glucose or glycated haemoglobin.

Serology screening for EBV, hepatitis B and C, HIV, Lyme, and other tick-borne diseases may be useful according to clinical and epidemiological features [40].

Other tests may be required according to availability of resources or as clinically guided. These are usually reserved for specialist centres or are done through referral to other specialties. These are usually aimed at differential diagnosis but could also be used for better characterization of pathology or for the assessment of function and disability (Box 6). Examples include anti-CCP, transglutaminase antibodies, morning cortisol, vitamin B12, NT-pro BNP, and vitamin D3 or 25(OH)D. In some cases, an extended auto-immune screening, allergy testing, serum tryptase levels, and/or lymphocyte differentiation may be required. Imaging and other specialised tests may be appropriate in some cases but are usually reserved for specialist centres, e.g., brain or spine MRI, cardiopulmonary exercise testing (CPET), cognitive testing panel, echocardiography, and tilt table or standing test.

Tests results will often be unremarkable, though subtle abnormalities may be observed [40]. Routine inflammatory markers are usually not elevated in ME/CFS. Low CK suggests severe disease or very low physical activity levels [41]. Elevated LDH and GPT/GOT are found in a subset of patients. Elevated NT-pro BNP might be found and is associated with lower cardiac volume [42]; this should be investigated further. A subset of patients has diminished IgG/A/M levels and/or IgG subclass deficiency [43]. Marked abnormalities should raise the suspicion of an alternative diagnosis.

Box 8. **Core and additional assessments that may be recommended for ME/CFS secondary care services.**

**Domain or Specific Clinical Situations**

**Clinical, Laboratory, and Imaging Assessments or Measurement Instruments**
CORE ASSESSMENTSSeverity assessmentUKMEB-PQsymp; DPQ, RAND-36, Pain and fatigue analogue scales Disability screeningRAND-36 summary scales (physical and mental component summaries) Muscle power and general healthHand grip measurements, dynamometer ADDITIONAL ASSESSMENTSRoutine tests not done recently and justified clinically Tests as appropriateIf clinical history suggests autoimmune or immunodeficiencyANA, ENA, TPO, AMA, APA, immunoglobulins, and others according to clinical findings Serious neurocognitive symptoms that increase risks for patientsNeurocognitive tests—e.g., Creteil battery of tests; NIH CDE Toolbox (National Institute of Neurological Disorders and Stroke (NINDS), 2018) [43] Neuroimaging as needed for further neurological investigationsMRI scan, CTObstructive sleep apnoea suspectedSleep studies, polysomnography Signs of small fibre neuropathy, peripheral neuropathy, marked muscle symptoms, objective peripheral findings Nerve conduction studies, electromyography (EMG), skin (for intradermal nerve fibre density) or muscle (rarely necessary) biopsyPOTS, orthostatic intoleranceTilt table test or repeated recumbent and standing heart rate and blood pressure (standing test) Objective assessment of PEM or disability 2-day CPT (use with caution as can cause or aggravate PEM)Other more recent tests which may be useful Metabolomics, e.g., those revealed through organic acid testing and amino acid urine and serum, cytokine panels, and autoantibodies to receptors such as adrenergic receptors.* A selection or the full range of tests may be conducted routinely or in support of disability assessment. AMA: anti-mitochondrial antibody. ANA: anti-nuclear antibodies. APA: anti-phospholipid antibodies. CPT: cardio-pulmonary testing. DPQ: DePaul Symptom Questionnaire. ENA: extractable nuclear antigens. PEM: post-exertional malaise. POTS: postural orthostatic tachycardia syndrome. TPO: thyroid peroxidase. UKMEB PQsym.: UK ME/CF Participant Questionnaire.

### 3.5. Steps to Recognising ME/CFS in Children

None of the criteria used in adults have been validated for the diagnosis of paediatric ME/CFS. Diagnosis of ME/CFS in children is especially challenging for two main reasons: First, younger children may not report symptoms accurately and might assume fatigue as normal, when not remembering the experience of full health. Second, there are differences in how children perceive and report symptoms of ill health, and proxy reporting by parents may not always accurately reflect children’s experience. To account for the latter, paediatric ME/CFS should be diagnosed if CCC are fulfilled for as little as 3 months and no other underlying disease has been identified (Box 2). Owing to differences in manifestations and their ascertainment in children, compared to adults, a paediatric case definition that uses the structure of the CCC 2003 adults’ definition and some aspects of the CDC-1994 criteria was published in 2006 [30] and modified in 2018 [5]. Most recently, a group of experienced paediatricians suggested a “Clinical Diagnostic Worksheet” [4] (Box 4). This guidance refers to “impaired function” or a “substantial reduction in the child’s ability to take part in personal, educational, and/or social activities” associated with fatigue and PEM as cardinal symptoms. Other symptoms including headaches, myalgia, joint pain, sore throat, painful lymph nodes, and abdominal pain are scored as “pain” [4].

Symptoms usually start acutely, often following symptoms of infection, e.g., flu-like symptoms, or gastroenteritis, but may have an insidious or episodic onset. In children, about half of the cases of ME/postinfectious fatigue syndrome manifest after typical Epstein–Barr-virus (EBV)-associated infectious mononucleosis [44,45,46]. Symptoms are usually fluctuating in type and severity (especially in the early stages of the disease), with patients typically reporting “good” and “bad” days. A more careful analysis of the pattern of symptoms may reveal correlation with physical or mental efforts.

Primary care professionals may suspect a diagnosis in children and adolescents presenting with persistent or recurrent moderate to severely impaired function, fatigue, and post-exertional symptoms, especially if associated with autonomic symptoms, sleep disturbance, neurocognitive problems, and pain (e.g., headaches and abdominal pain), following history, clinical examination, and routine tests that exclude other diagnoses that may explain the symptoms. We recommend paediatricians use the full criteria from Rowe et al. (2017) [4] as part of diagnostic approach and the CCC 2003 criteria [2] if symptoms are present for 3 months.

### 3.6. Diagnostic Categories

A proposed diagnostic characterization of patients, which builds on previous disease criteria definitions, is shown in Box 9, which also suggests stratification variables that may be used for sub-grouping of cases.

Box 9 **Diagnostic categories and sub-grouping.**

**Symptom description** ***Prolonged fatigue:*** persistent profound fatigue or lack of energy, usually (but not necessarily) accompanied by other symptoms; should be present for at least one month ***Chronic fatigue (CF):*** persistent fatigue or lack of energy, that leads to reduced activity levels lasting over 3–6 months*. This may be explained by a condition other than ME/CFS (e.g., cancer-related fatigue) or unexplained (“idiopathic chronic fatigue”). It does not require other symptoms that are typically found in ME/CFS ***Post-infectious fatigue or post-viral illness (PIF or PVI):*** new onset symptom complex including persistent profound fatigue with exercise intolerance following an infectious trigger and which is not otherwise explained by a diagnosed condition or lifestyle. It is usually accompanied by at least 2 further symptoms** from: post-exertional malaise, unrefreshing or poor sleep quality, cognitive or autonomic symptoms for at least 3 months (i.e., this is a subset, where the viral aetiology is clear, of patients with chronic fatigue). **Diagnostic categories**
***ME or ME/CFS:*** persistent fatigue or lack of energy that leads to reduced activity levels lasting over 3–6 months, when diagnostic criteria according to IOM or Canadian Consensus criteria (CCC) are fully met for adults, and CCC or Rowe’s criteria are fully met in children.***ME/PVFS (ME/Post-viral fatigue syndrome or post-infectious fatigue syndrome, post-infectious ME/CFS):*** As for ME/CFS, when symptoms follow a presumed or confirmed infection (e.g., post-COVID-19 fatigue syndrome, post-mononucleosis fatigue syndrome, post-Lyme ME/CFS) (NB. This does not preclude there being triggers other than infections involved in the origins of the illness in other cases)***Non-ME chronic fatigue:*** chronic fatigue cases that do not fulfil the diagnostic criteria for ME/CFS, lasting for at least 3–6 months, but are attributable to other underlying causes.***ME/CFS of combined aetiology:*** when symptoms are attributed to a combination of ME/CFS and other known disease(s), e.g., ME/CFS and diabetes type 2 (NB. This is not in itself a diagnosis, which requires identification of the disease(s) to which the condition is attributable). 
**Examples of stratification categories:**
Age-group (e.g., children, adolescents, adults, elderly), gender Illness onset: acute or gradual; post-infection, following other triggers, e.g., environment exposure Presence of co-morbidities, e.g., fibromyalgia, hypermobility, mild mood disordersPhase of disease (or disease duration), e.g., early, established, and complicated disease (Nacul et al., 2020) [7]Severity (based on symptoms score or measures of function); a broad categorisation of severe/non-severe is based on being virtually house-bound or able to regularly be outside home. Very severe cases are virtually bed-bound.Clinical phenotype: based on predominance of symptoms by type (e.g., based on CCC symptoms sub-groups); e.g., neuro-cognitive, immune, sleep phenotypes (NB. There are distinct clinical phenotypes in ME/CFS which can be identified from gene expression data [47,48]). One study identified seven genomically derived subtypes of ME/CFS which manifested distinct phenotypes [49,50].Molecular phenotype: i.e., based on well-defined profiles based on results of specialised investigations, e.g., metabolic, immunological. 
* CCC 2003 [2], IOM 2015 [6], and Rowe et al., 2017 [4], criteria require 6 months of symptoms; experienced clinicians should be able to diagnose adults with 3 months of symptoms. For children, CCC criteria requires 3 months [2], and Rowe et al., 2017 [4], require 3 months in post-infectious cases. ** The 2 additional symptoms criterium is not required when the fatigue symptoms can be clearly linked to the triggering infection and are not explained by other pathologies.

Chronic fatigue-spectrum disorder (CFSd) is an encompassing term and may be used to refer to persistent profound fatigue for over 3–6 months associated with other symptoms, including the following sub-categories: (a) cases meeting diagnostic criteria for ME/CFS; (b) cases that do not fully meet diagnostic criteria (Non-ME chronic fatigue-Sd) but cannot be explained otherwise; (c) cases totally or partially explained by other diseases known to cause chronic fatigue (disease-associated CFS; or ME/CFS of combined aetiology).

### 3.7. Recommendations for Health Care provision

Primary care professionals have an important role in the initial diagnosis, including consideration of alternative conditions leading to similar symptoms. It is important to note that many symptoms commonly reported in ME/CFS have a low disease-specificity and may occur in a number of diseases. Acute infectious onset and PEM should always prompt to consider ME/CFS. Although diagnostic confirmation may require a 3- to 6-month period, it is important to contemplate the diagnosis at earlier stages, so that disease management may start, and diagnosis and treatment of alternative diseases are not delayed (See Box 7).

Careful medical history, including social and occupational history and circumstances associated with the start of symptoms and subsequent progress will give significant clues on diagnosis. Information should be obtained on current and previous treatments, including prescribed and over the counter medicines and supplements as well as self-management strategies and alternative therapies. It is important to check for medications potentially leading to fatigue as well as autonomic-related and other symptoms. Physical examination and routine bloods tests are required to increase diagnostic accuracy and detect alternative conditions explaining the symptoms [51].

Patients with ME/CFS tend to be multi-symptomatic and often have long clinical histories, which may include various failed attempts to obtain a diagnosis and treatment. Multiple previous investigations are not uncommon; however, often, symptoms presented are discarded by clinicians as “exaggerated” or “imagined”, related to excessive work or studies or as mood-related. Such a scenario is to be avoided through early recognition and diagnosis, which are reliant on better knowledge of the disease and education of doctors and other health professionals.

When a diagnosis is suspected in primary care, regular reviews are warranted, when the possibility of alternative diagnoses is explored at the same time as initial management, strategies are put in place. In such cases, it may be helpful to ask the patient to record their symptoms and other health parameters using standard instruments in advance of follow-up consultations (see Core Assessments, Box 8).

Education of patients in advance of, during, and following consultation may be useful, and reliable educational materials should be recommended, e.g., booklets, videos, or other online information materials. These should cover concepts and practical recommendations for “pacing” (pacing is a self-management tool to implement a strategy designed to help people live within their energy envelope, minimise PEM, and improve quality of life [52]) with adequate rest periods or breaks in activity, sleep hygiene, and pain management strategies. Both mental and physical activities should be taken in such a way to avoid over-exertion, which may trigger post-exertional aggravation of symptoms or “crashes”, and as a key strategy to optimise chances of recovery. A main goal of educational activities is to empower the patient for self-management and to be in control of their disease and healing process.

### 3.8. Criteria for Referral for Specialist Services

Although with good education of primary care physicians, diagnosis and monitoring of people with ME/CFS in primary care are possible and desirable, and referral for specialist services may be indicated in some circumstances (Box 10), viz. for confirmation of diagnosis, when there is doubt; for cases who may benefit from a multi-disciplinary team with specific expertise, including drug treatments or care of those with severe or complicated disease; and for a range of service offerings, such as occupational therapy, supportive counselling, education on self-management and energy/activity management with “pacing”, social services, and advice on access to community support, e.g., for educational, occupational, and social matters, such as benefits (see below on secondary services). Patients with more recent disease onset, such as those with less than 1–2 years of symptoms and the young (children, adolescents, and young adults) may also benefit from referral for initiation of multicomponent therapy, as early referral at this age might especially affect long-term prognosis. The more severely affected, including those who are house- or bedbound and severely disabled, should also be priority for referral, especially where appropriate home-visits or telemedicine are available, and, when necessary, for occupational, educational, and disability support. Note that some cases may be best served by referral to alternative services, especially where ME/CFS or Complex Chronic Diseases (CCD) Services are not well developed, such as to pain management, rehabilitation, neurology, psychiatry, and rheumatology services.

Box 10 **Examples of criteria for referral to secondary services caring from people with ME/CFS.**

Diagnosis confirmation Young people Severe cases or significant disability, especially if local support is limited Short duration of symptoms (less than 1 or 2 years) Rapid deterioration of symptoms Complex diseases, where diagnosis and treatment are challenging Inability to provide adequate care in the community or when management and treatment are only available at specialist services

### 3.9. The Continuing Role of Primary Care and the General Practitioner

In general, irrespective of referral to secondary care, whenever possible, the primary care team should continue to take responsibility for the long-term care and monitoring of patients with ME/CFS and their treatment, whenever possible in partnership with the specialist team. This includes facilitating the provision of emotional, social care, and occupational health support, and medical advice to teachers, employers, and caregivers, in response to the specific needs of patients. This could involve access to resources in the community, such as to physiotherapy, occupational therapy, dietician, or home visits by the primary care team (especially for the more severely affected), e.g., by district nurses. Support for self-management, education, and work activities may require further contacts with the patients and their carers/families, as well as with educators and employers. Here, online educational materials may be of value, as well as group educational activities for patients. Organization of care for people with ME/CFS and in particular the severely affected may be complex and requires communication by primary care professionals with others from various disciplines.

The primary care provider will still have major responsibilities for searching for alternative diagnoses where relevant and dictated by clinical judgement, for dealing with co-morbidities, the onset of new co-morbidities, and other diseases that may be not directly related to the diagnosis of ME/CFS, and for referring to different specialists as appropriate. Pharmacological and non-pharmacological approaches to treatment and clinical progress should be reviewed. It is important to consider that patients with ME/CFS may be more sensitive to a range of medications; this also needs to be considered when treating other conditions, having in mind also the possibility of drug interactions.

Needless to say, the strength of primary and secondary care services in particular settings will be relevant to determine roles at each care level and the best ways of cooperation between services at different levels. We appreciate limitations of access and service provision in primary care in many places, and local solutions will need to be found in line with local needs and resources. Virtual healthcare or virtual support from the specialist to the primary care team may have an important role.

## 4. The ME/CFS Specialist Consultation

### 4.1. Preparing for the Consultation and the Waiting Room

Before specialist consultation, it may be helpful to obtain relevant information, using standardised questionnaires or data/information otherwise obtained that may help with diagnostic confirmation, characterization of symptoms and their severity, and life impact. Forms may also be used as baseline clinical information for monitoring disease progress and response to management or treatment. These can be completed before consultation.

Standard questionnaires include the UKMEB Symptoms Assessment Questionnaire (SAQ) [53], to aid diagnosis and the Participant Phenotyping Questionnaire (PPQ), for severity profiling [54] or the DePaul Symptom Questionnaire, allowing diagnosis and symptom severity profiling [6]. The Impact on function and quality of life may be measured by standard instruments, such as Rand-36 [55,56], some of which have been validated in many languages. The Epworth Sleepiness Scale [57] can be used to assess excess daytime sleepiness and as a screening for obstructive sleep apnoea. Other instruments may be used to screen for mood disorders, e.g., neuroQOL [58] or HADS [59] for depression and anxiety or GAD-7 [60] for anxiety. Fatigue severity may be measured by instruments validated for ME/CFS, e.g., the fatigue severity scale [61]; visual scales such as pain and fatigue analogue scales are simple to use [62,63]. The same applies to sleep disorders (e.g., the Pittsburgh Sleep Quality Index [64]), and autonomic symptoms (e.g., Compass 31 [65]. A diagnosis of fibromyalgia may be established with a good degree of confidence by the annotation of pain symptomatology in pictorial representation of the human body [66]. The same is true for the evaluation of hypermobility syndromes, using the Beighton criteria [35].

### 4.2. Diagnosis Confirmation and Continued Search for Alternative Diagnoses and Co-Morbidities

The list of differential diagnoses of fatigue is exhaustive. Examples are listed in Box 7. Some conditions are often present concomitantly to ME/CFS (co-morbidities). Other conditions may potentially exclude a diagnosis if they may fully or mainly explain the symptoms. However, such conditions may also be co-morbid when their presence does not explain most of the symptoms and signs observed.

For diagnosis confirmation, we recommend the use of the CCC in both adults and children [2]. Additional tools for adults include The IOM criteria [6] and for children the paediatric “Diagnostic Work Sheet” [4] and/or the DSQ-PED [5]. Full consideration needs to be given to differential diagnosis and co-morbidities, and the need for detailed history, physical examination, and complementary tests, as appropriate, cannot be overestimated. Further tests may be recommended in secondary care settings, according to the need for supporting ME/CFS diagnosis and/or severity, and for differential diagnosis. Box 8 lists some assessments that may be considered. Those marked are suggested to be run routinely at the first assessment, and the others should be evaluated on a case-by-case basis, based on the clinical presentation. RAST tests for specific allergies, echocardiography, and serology for specific infectious diseases, as guided by clinical and epidemiological information, are other modalities that may be considered as appropriate.

Treatment for children and young people should usually be started by a paediatrician or a ME/CFS secondary care specialist centre that includes a paediatrician.

Further referral may be required when alternative diagnoses are suspected. This may include referral to a neurology or multiple sclerosis (MS) clinic and/or to specialists in ophthalmology, ENT, immunology (autoimmunity, immune dysfunction), allergology, orthopaedics, physical therapy, infectious diseases (travel-related disease), psychiatry, or gastroenterology.

### 4.3. Management and Treatment

In the absence of disease-specific treatment, key roles of the health professional include confirming the diagnosis, explaining to the patient the importance of avoiding overexertion and mental stress, “pacing”, and symptomatic medication as needed and appropriate for the patient. Regular monitoring is important, when progress should be assessed, and the possible development of new diagnoses and co-morbidities considered, as the management plan is reviewed. “Pacing” refers to breaking up physical or mental activities with periods of rest, before a significant level of tiredness or exacerbation of symptoms is achieved or is expected following exertion, e.g., PEM, which may manifest many hours after the effort. A general rule of thumb is the recommendation to keep the activity at 2/3 of the duration and of the intensity that is expected (based on previous experience) to cause post-exertional symptoms, though flexibility should be exercised in order to reflect the particular needs and circumstances of individual patients.

The goal of the management/treatment programme is to treat the most distressing symptoms (sleep disturbance, pain, orthostatic intolerance, or others) and empower the patients to be in control of symptoms and the disease by encouraging them to trust their own experiences and enhance their awareness of the activities and environments in which they can cope without exacerbating symptoms, and “pace” themselves accordingly. The program should aim at optimizing the patient’s ability to maintain function in everyday activities, being as active as possible within their boundaries and then gently extending those boundaries [2]. This may be challenging, especially in the more severely affected who may be able to tolerate only very low levels of activity; those with less severe forms of disease are likely to “overdo” and may have frequent exacerbations of symptoms (“crashes”) as a consequence.

Recent studies suggest that there may be a role for cognitive behaviour therapy (CBT) in the management of ME/CFS. It may have long-term benefits in chronic fatigue [67], but there is little evidence of this, and it needs to be used with considerable care to avoid distress [68]. It should be appreciated that it is a supportive therapy and not curative [69].

Wearables can assist objective measurement of activity and sleep patterns, and in some cases heart rate variability [70]. They may be combined with a symptom diary, which will help the interpretation of symptoms and management.

### 4.4. Professional-Patient Partnership, Self-Management, and Support

It is important to establish a supportive and collaborative relationship with the patient suffering from ME/CFS and, as appropriate, with their caregivers. Engagement with the family may be essential, especially for children and young people, and for people with severe ME/CFS. A named healthcare specialist should be involved for coordinating care for the person with ME/CFS. Information to people at all disease stages should be according to the person’s circumstances, including clinical, personal, and social factors. Information should be available in a variety of formats as appropriate (printed materials, electronic videos, and audios).

The doctor-patient partnership, informed choices and risk minimisation are essential components of care. Partnership between patient and health care providers should be based on trust, and consideration of their interactions as encounters between two experts with different, but complementary backgrounds (the patient and the healthcare provider), who recognise that knowledge about the disease and its management is incomplete. Basic management principles should apply, but often different treatments may be attempted (preferably one at a time, on a trial-and-error basis) and reassessed according to response or potential adverse effects. This is when the strength of the partnership becomes even more important, as partners engage in a journey where uncertainty is gradually replaced by increasing understanding of the disease/health process, as treatment and management strategies are regularly reviewed and adapted to suit patient characteristics and preferences. Over-investigation and over-treatment are discouraged, but a very passive approach to illness may also be counterproductive, and such discouragement should not result in patients being denied treatment or testing needed to monitor changes in their condition.

### 4.5. Managing Patients’ Expectations

It is essential that the professional is upfront in explaining the current limits of treatment and understanding of potential pathophysiology and the approach to symptom management. This will greatly address discrepancies between patients’ and doctors’ expectations and set up the conditions for an open and positive patient-doctor relationship where patients are empowered to make informed choices.

There is no known pharmacological treatment or cure for ME/CFS. However, symptoms should be managed as in usual clinical practice. Physicians may consider starting symptomatic treatment at a lower than usual dose, due to frequent medication sensitivities in this population. The dose may be carefully increased. Treatment and repeat prescription may be continued in primary care, depending on the patient’s preference and local circumstances.

### 4.6. Non-Pharmacological Treatment for Symptoms Relief and Available Support Therapies

Recommendations considered appropriate are shown in Box 11. It is important that these are provided by practitioners with experience in ME/CFS.

Box 11 **Recommendations for a non-pharmacological approach to the relief of ME/CFS symptoms.**

***Pain***
RelaxationMeditation/mindfulnessManual methods (e.g., physiotherapy, acupuncture, and acupressure)
***Sleep***
Sleep hygieneRelaxation strategies
***Autonomic dysfunction, e.g., POTS***
StockingsIncrease in water intake (>2 litres/day) or rehydration solutions, drinking frequentlyIncrease in salt intakeSleep with feet in higher position (a few centimetres higher, increasing very slowly each night, up to what is tolerated)
***Diet***
Healthy and balanced dietAnti-inflammatory dietReduce ingestion of simple carbohydratesAdequate fluid intakeAdequate ingestion of proteinIncrease unsaturated fatty acids and omega-3 fatty acidsMay try exclusion diets with support from dietician, especially for food with reported intolerances by the patient. It may be worth trying to avoid gluten, lactose, or fructose during a few weeks to test if there is any improvement in symptoms [71].
***Support measures***
“Pacing” and activity management to work with the “energy envelope” [72] Supporting therapies that could help with coping and adapting to changes in life due to symptoms, within the “energy envelope”, and counselling or psychotherapyOccupational therapy provided by professionals with experience in ME/CFS patientsSocial workers who could help with social welfare Educational needs: welfare and educational sectors should be involved in the planning and care for affected patients, particularly children, adolescents, and young adults

***A professional view on symptom management and relief***
*“Periods of rest and “pacing” are important components of all management strategies for ME/CFS patients. Physicians should advise people with ME/CFS on the role of adequate rest, how to introduce breaks into their daily routine, and their frequency and length which may be appropriate for each patient. Excessive rest may be counterproductive, except in the initial stages of disease, in the very severe cases, or in cases of acute exacerbation; so it is important to introduce ‘low level’ physical and cognitive activities within the patient’s capacity, according to the severity of symptoms.* *Sleep management is tailored to the individual, the role and effect of disordered sleep is explained, common changes in sleep dysfunction that may exacerbate fatigue symptoms are identified; common manifestations include insomnia, hypersomnia, sleep reversal, altered sleep-awake cycle and non-refreshing sleep. The professional provides general advice on good sleep hygiene and encourages gradual changes in sleep pattern, though of course there is no implication that poor sleep hygiene is the cause of non-refreshing sleep. Relaxation techniques appropriate for ME/CFS should be offered for the management of pain, sleep problems and comorbid stress or anxiety. Examples include guided visualisation and breathing techniques, which can be incorporated into daily routines and rest periods”, while mindfulness ma be of value as a sympathetic nervous system modulator. Although exclusion diets are not generally recommended for managing ME/CFS, many people find them helpful for some symptoms, including bowel symptoms. The patient may attempt an exclusion diet or dietary manipulation under professional guidance and supervision, e.g., from a dietitian. For those with nausea, advice includes eating small portions and snacking on dry starchy food and sipping fluids. The use of anti-emetic drugs should be considered if the nausea is severe.”* Dr. L. Lorusso (personal communication)

### 4.7. Symptoms Relief and Management Using Available Pharmacological Drugs

Treatment of pain and sleep dysfunction are key, as they may have an indirect impact on other symptoms. Options for the pharmacological treatment of fatigue, including mental fatigue are more restricted. A balance between benefits and side effects, and significant individual variability in treatment response, call for individualised treatment. Costs are also a consideration, especially in settings where patients pay for medications out of pocket or where there are restrictions in prescribing medications.

Evidence of the effects of various drugs or supplements is scarce and often based on their use for related conditions or on reported use in ME/CFS and clinicians experience. It is important to observe legislations in different countries and to ensure that prescription of any drugs not specifically approved or licensed for ME/CFS is discussed with the patient and an informed choice is made. In some settings, it may be appropriate to obtain formal signed consent from the patient before introduction of a drug that has not been approved for use in ME/CFS. Regulations on supplements and over the counter medications are usually much less strict, but again, use by patients should be based on informed decision. Finally, it is important to note that many patients have already been taking a range of medications and supplements before reaching the ME/CFS specialist; again, in these cases, it is important to discuss continuation or otherwise with the patient; evidence of benefit on the individual patient, costs, potential side effects, or interactions with other medicines are important considerations. Some examples of pharmacological drugs that could be considered, where appropriate, are listed in Box 12. The use of medications that may address multiple symptoms may be considered.

Box 12 Examples of pharmacological approaches for relieving/managing ME/CFS symptoms*.

***Pain***ParacetamolNSAID (for short periods, e.g., up to 7 days)Gabapentin or pregabalinTricyclics, such as amitriptylineLow dose naltrexone DuloxetineVenlafaxine***Sleep***Tricyclics, e.g., amitriptylineTrazodoneMelatoninDoxepin low doseDiphenhydraminePromethazineBenzodiazepines and Z-drugs (for short periods only)Gabapentin/pregabalin***Autonomic dysfunction, e.g., POTS***FludrocortisoneSSRIMidodrineIvabradinePyridostigmine***
Anti-allergic/anti-inflammatory***Antihistamines, e.g., fexofenadine or famotidineSodium cromoglicate**Supplements** which may be tried for symptoms such as fatigue or cognitive dysfunction
Iron (if ferritin < 50 ug/l, transferrin saturation <20%)Vitamin DL-carnitine or acetyl-carnitineCoQ-10 or MitoQNADHVitamin B12.α-lipoic acidMagnesiumOmega-3 or omega-3/omega-6 combinationD-RiboseVitamin B1, B2, and/or B6Vitamin C* Refer to local guidelines on the use of medications that are not specifically licensed for use in ME/CFS.

### 4.8. Following the Consultation and Clinical Monitoring

Regular follow-ups are opportunities for education, including on self-management, assessment of usefulness of medications and other treatments and side-effects. Follow-up should include monitoring of symptoms, using similar instruments to those used at or before the initial consultation. Examples of instruments that may be used in monitoring patients include hand grip strength measurement, standing test, serum CK, severity assessment using specific instruments or scales (such as analogue scales for pain, fatigue, sleep, and other symptoms), and specific questionnaires for assessing symptom severity.

### 4.9. Needs of Patients with Different Severities

People who have severe ME/CFS may be unable to carry out activities of daily living and may spend a significant proportion, or all, of the day in bed. The symptoms experienced by patients with severe ME/CFS are diverse and debilitating, and these may fluctuate and change, both in type and in severity. It is therefore important that the management and care plan is flexible and reviewed regularly. People may have severe ME/CFS for years, and recovery is uncertain. Health services need to be prepared to attend to the specific needs of the severely affected, including home visits or virtual health consultations.

## 5. Concluding Remarks and Recommendations for Developing and Organising ME/CFS Services

The following are general recommendations for fully implemented services, but we appreciate that they are not achievable in the short term in many places, especially where knowledge and training in the field are limited or other resources are scarce. We encourage countries and regions to plan for their services, training, and educational needs according to the specific needs and characteristics of their population and patients and their organizational structures and resources. A national champion for each country or regions within countries would be highly desirable, especially in places with no or very scarce provision of services for ME/CFS.

For fully functioning services, we recommend 2–4 ME/CFS specialist doctors/1 million population, with a supporting multi-disciplinary team, to include professionals such as nurses, nurse practitioners, occupational therapists, psychologists, dieticians, social workers, etc.; these would staff outpatient services for diagnosis and follow up. The specialist may be a doctor with expertise in ME/CFS. Internists, neurologists, immunologists, rheumatologists, infectious diseases specialists, and general practitioners are particularly suited for this role, but it may be done by doctors of any specialty, as long as they have the right expertise or training. For children, this role is to be filled by paediatricians. At the time of writing, we are not aware of any specific programme for the training of doctors to become specialists in ME/CFS, something that has often occurred informally so far. The training and provision of services in secondary care should be aligned with the training of primary care physicians to manage cases in the community. We recognize that the above target is ambitious, considering the current capacity and status of service provision in the continent. They should be seen as tentative and should not replace the assessment of patients’ needs and structure and capacity of services at local and national levels.

The current reality of health services suggests that, where specialist services are not well developed, we follow a minimum standard of care for those with ME/CFS that may rely on virtual health and app-technology as well as on a strong partnership with primary care.

The minimum desirable is one ME/CFS centre providing specialist services for a 10 million population. These services should also consider the characteristics of the population, including ethnic and cultural diversity. Furthermore, we recommend that the specialist services should have the primary aim of confirming diagnosis and setting up treatment/management plans, which should be agreed upon and carried out by a multidisciplinary team. The follow-up could use multi-media approaches, such as remote consultations or telemedicine, as appropriate according to local circumstances and medical regulations. Local care for people with significant disability may need to be provided by primary care teams or local doctors with knowledge about ME/CFS, with support from the specialist services as appropriate. The option of smaller satellite clinics linked to the specialist service would provide full assistance for most and the “eyes” of a competent health professional, in support of remote consultations from the specialist for complex cases.

There is no suggestion that people with ME/CFS require more social support than people with other chronic diseases, and we are most certainly not implying that the disease is primarily psychological in nature. We are, though, very well aware that people with other chronic diseases, such as for example diabetes or multiple sclerosis, do not have the same problems of disbelief and lack of legitimisation experienced by people with ME/CFS. All people with chronic diseases need, and should be entitled to, social support, but few experience the same difficulty accessing it as people with ME/CFS.

Finally, it is important to consider that addressing the substantial needs of people with ME/CFS requires a multi-sectoral approach (Box 13), as well as ensuring that health services are organised and delivered effectively. Much of the needs of people affected by ME/CFS arise from their reduced ability to function in society and in more extreme cases on their total dependence on care for basic needs. Work, life, and education may be disrupted, with substantial economic and personal impacts on individuals and their families; lack of understanding and support, and often stigma, adding to the burden of physical suffering from symptoms. It is extremely important to prioritize research and education of health professionals and others in society, so as to address the scientific and societal poor understanding of the scale of the problem faced.

Box 13 **Multi-sectoral approach to ME/CFS.**

**Specific societal sectors** Higher education: Development of training for under-graduates and post-graduates, including training for primary care staff and occupational physiciansEducational sector: Development of materials for teachers and education staff, as well as for pupils with ME/CFS and their parentsWork and pensions: Development of adequate instruments for assessing disability and flexibility in workplaces, particularly after returning to work, to minimise the risk of relapseHealth sector and public health:Adoption of guidelines, flexibility on the use of medications for management of symptomsPublic health strategy for raising awareness about stigma, importance of care and education to avoid aggravation of symptoms and/or relapseME/CFS services development and evaluationFunding agencies and pharmaceutical industry: Research funding and support for well-designed clinical trials

## Data Availability

No new data were created or analysed in this study. Data sharing is not applicable to this article.
